# Validity of Quinpirole Sensitization Rat Model of OCD: Linking Evidence from Animal and Clinical Studies

**DOI:** 10.3389/fnbeh.2016.00209

**Published:** 2016-10-26

**Authors:** Ales Stuchlik, Dominika Radostová, Hana Hatalova, Karel Vales, Tereza Nekovarova, Jana Koprivova, Jan Svoboda, Jiri Horacek

**Affiliations:** ^1^Department of Neurophysiology of Memory, Institute of Physiology, Czech Academy of SciencesPrague, Czech Republic; ^2^National Institute of Mental HealthKlecany, Czech Republic

**Keywords:** OCD, quinpirole, animal model, brain circuits, human, rat

## Abstract

Obsessive-compulsive disorder (OCD) is a neuropsychiatric disorder with 1–3% prevalence. OCD is characterized by recurrent thoughts (obsessions) and repetitive behaviors (compulsions). The pathophysiology of OCD remains unclear, stressing the importance of pre-clinical studies. The aim of this article is to critically review a proposed animal model of OCD that is characterized by the induction of compulsive checking and behavioral sensitization to the D2/D3 dopamine agonist quinpirole. Changes in this model have been reported at the level of brain structures, neurotransmitter systems and other neurophysiological aspects. In this review, we consider these alterations in relation to the clinical manifestations in OCD, with the aim to discuss and evaluate axes of validity of this model. Our analysis shows that some axes of validity of quinpirole sensitization model (QSM) are strongly supported by clinical findings, such as behavioral phenomenology or roles of brain structures. Evidence on predictive validity is contradictory and ambiguous. It is concluded that this model is useful in the context of searching for the underlying pathophysiological basis of the disorder because of the relatively strong biological similarities with OCD.

## Introduction

Obsessive-compulsive disorder (OCD) is a chronic, heritable and debilitating neuropsychiatric disorder with a lifetime prevalence of about 1–3% (Stein, 2002). The etiology of OCD remains unclear. It has been shown that approximately 47% of the variance in OCD is explained by genetic factors and the remaining variance depends on environmental factors (van Grootheest et al., 2005). Commonly discussed potential causes include the pathophysiological effects of infections and stress and traumas in childhood. These influences have significant effects on the central nervous system, specific anatomical circuits and neurotransmitter systems.

Scientists widely use animal models to understand the disease mechanisms and search for new therapies. An animal model can barely mimic the entire disease in all its aspects. The animal models are considered tools for evaluating mechanisms which may play the role in particular disease rather than “mini-versions” of the disease in animals. Evaluation of aspects linking animal model and disease is based on a concept of models in general (e.g., Rosenblueth and Wiener, 1945). This concept was adapted to modeling of neuropsychiatric diseases in animals by Willner (1986). Sets of criteria in which an animal model represents components of the disease are called axes of validity. These can be classified into three categories: face, construct and predictive axes of validity (Willner, 1986). Face validity corresponds to homologies seen in behaviors as the main phenomenological outcomes observable both in model species and humans. Construct validity corresponds to involvement of the same neurophysiological mechanisms and changes in the brain circuits (Albelda and Joel, 2012). Predictive validity corresponds to the predictability of treatment outcomes, or in a more general view, to the power of a given model to explain the clinical findings. This division is still useful in describing animal models, although some refinements in these criteria have been presented as well (Belzung and Lemoine, 2011).

Animal models of OCD can be classified into three main types: genetic, behavioral and pharmacological models. They have been reviewed in several excellent works (e.g., Eilam et al., 2006; Albelda and Joel, 2012; Alonso et al., 2015; Szechtman et al., 2016). This review aims at discussing and evaluating axes of validity of a model based on behavioral sensitization of rats to quinpirole. This model is characterized by compulsive checking, a form of rituals present in one of the symptom dimensions in OCD. The essentially pharmacological nature of this model allows the study of brain substrates underlying these behavioral changes and to directly compare them with a clinical situation. There is relatively extensive neurobiological evidence on this model. This fact allowed us to evaluate in detail it validity, focusing on the aforementioned three main axes.

## Quinpirole Sensitization Rat Model (QSM)

An established pharmacological animal model of obsessive-compulsive disorder is based on chronic administration of quinpirole, an agonist of dopaminergic D2 and D3 receptors. This treatment induces behavioral sensitization to quinpirole and compulsive-checking behavior that is phenomenologically similar to human compulsive checking rituals. Its potential to model compulsive-checking symptoms of OCD was described in detail by Szechtman et al. (1998), but quinpirole effects on animal behavior had been studied already in older works with ethological and behavioral-pharmacology approaches (e.g., Eilam and Golani, 1989). These studies showed route-, but not-movement-related stereotypical patterns. Origins of the ethological criteria for defining these patterns in the quinpirole sensitization model (QSM) come from earlier studies by Eilam and Golani (1988, 1989).

## Face Validity of the QSM

### OCD Phenomenology

OCD is characterized by recurrent thoughts (obsessions) and repetitive behaviors (compulsions). Compulsions are often reported by patients to “neutralize” obsessions and reduce obsession-related anxiety (Stein, 2002). Obsessive and compulsive symptoms can be defined in several principal domains. It was proposed that they could be mediated by a deficit in behavioral flexibility, such as the ability to shift focus of attention. Indeed such deficits were demonstrated in specific task-switching and reversal learning paradigms (Chamberlain et al., 2005, 2006). Additionally disruptions of recognizing conflicting information (Ciesielski et al., 2011) can play a role. OCD has substantial negative effects on the patient’s quality of life, and carries a considerable socio-economic burden at the population level (Olesenn et al., 2012). OCD is a heterogeneous disorder and there are many different ways by which OCD can manifest in patients. There is also no specific age of onset or single etiology. Additionally, treatment responses and comorbidities in patients vary to a large extent.

### QSM Phenomenology

Quinpirole-sensitized animals exhibit excessive checking, and perseverative as well as altered reward-related behaviors. The QSM was discovered serendipitously during experiments with animal models of psychosis (Szechtman et al., 1994). It was its face validity that made researchers to consider it as an animal model of OCD. The uniqueness of this animal model lies in modeling OCD symptomology related to symmetry obsessions—compulsive checking (Mataix-Cols, 2006). Since it is challenging to directly compare human and animal behavior, a set of ethological criteria of compulsive behavior were devised (Szechtman et al., 1998). These criteria can describe both animal and human behavior in ethological terms: first, there are one or two particular (key) places/objects to which the subject returns more excessively than to other places/objects in the subject’s living space. Second, these particular places/objects are visited significantly more often than others. Third, a limited number of places are visited in between returns to the key places/objects. Fourth, a characteristic set of acts is performed at the particular places/objects. Finally, this set of acts is dependent on particular places/objects and change when the environmental properties of the places/objects are altered (Szechtman et al., 1998).

All these five criteria were shown to apply both to OCD patients and to the QSM (Eilam et al., 2012). This important article conceptualized these ethological criteria as translational tools for studying OCD patients. When a quinpirole-sensitized animal is repeatedly placed in an open field with several (usually four) objects placed at fixed locations, the rat generally directs its activity towards one or two objects and a “home-base”. Home-base is a place to which the animal returns most often (Szechtman et al., 1994, 1998, 2001). The animal thus visits excessively a relatively small number of available places. This is analogous to a patient who focuses on checking mostly the objects of his/her obsession during his compulsive rituals. If a home cage is placed in the open-field arena in which the rat conducts its checking behavior, it remains in the home cage for a while before coming out and resuming its checking activities again. Analogously, patients can usually withhold their checking behavior, but eventually they resume checking after some time.

Excessive perseverative behavior in the QSM animals has been shown in spontaneous alternation (SA) in a T-maze. SA is an intriguing phenomenon observable in many biological systems that has not been fully explained yet. During SA, intact animals enter a different arm of the T-maze in the next trial then on previous one. Quinpirole-sensitized animals alternate much less than control animals (Einat and Szechtman, 1995). This means that they show increased perseverative behavior similar to OCD patients (Yadin et al., 1991).

Quinpirole-sensitized animals also display abnormal reward-related behavior. They choose to work for a reward instead of choosing a freely available source (Jensen, 1963; Koffer et al., 1971). This phenomenon is called *contrafreeloading*. In excess, contrafreeloading is considered to be a manifestation of compulsive behavior (Amato et al., 2007; De Carolis et al., 2011). As an example of this paradigm, water-deprived animals are first trained to press a lever to receive a small amount of water. This part is called the operant conditioning phase. It is followed by a choice phase. Here animals can obtain water either by pressing a lever or directly from a freely available water container. Quinpirole-sensitized animals chose to obtain water significantly more by means of lever pressing than do saline treated animals. Also, quinpirole sensitized animals did not drink all the water they obtained. Actually they drank less water than saline treated animals (hypodipsia; Cioli et al., 2000; De Carolis et al., 2011; Schepisi et al., 2013). This shows that increased lever pressing was not driven by increased thirst.

Cognitive impairments, mainly at the level of behavioral flexibility are also considered intermediate phenotypes in OCD. Therefore, they could support the face validity of the QSM. Although sparse, there have been several studies observing cognitive impairments in the QSM. They were similar to those in OCD patients. As mentioned above, SA was found deficient in the QSM (Einat and Szechtman, 1995). Moreover, a significant deficit in the QSM was found in reversal learning (Boulougouris et al., 2009; Hatalova et al., 2014). Beside implications for OCD, these findings also suggest the involvement of D2/D3 receptors in these types of flexible behavior.

## Construct Validity of the QSM

### Neurobiological Changes in OCD

Despite the clear heterogeneity of OCD, there is a consensus in the involvement of the cortico-striatal circuits in OCD pathophysiology. Nonetheless, there is not yet a clear and complete answer as to which structures are altered in these circuits. There are structural (or volumetric), as well as functional changes. Structures that are associated with OCD include the orbitofrontal cortex (OFC), anterior cingulate cortex (ACC), prefrontal and parietal cortices, the caudate nucleus (Menzies et al., 2008), and *nucleus accumbens* (Figee et al., 2013b). These findings are consistent with most circuit models of OCD (Saxena et al., 1998; Menzies et al., 2008; Rotge et al., 2008) which propose that the pathophysiology of OCD reflects a dysfunction in the neuroanatomical network of cortical-basal ganglia loops described by Alexander et al. (1986).

### Neurobiological Changes in the QSM

The construct validity of the QSM is supported by the involvement of D2 dopamine receptors and the involvement of the striatum and the OFC. Following quinpirole sensitization, changes in striatal structures have been observed in the QSM. An increase of D2 receptor binding (Culver et al., 2008) and decrease of glucose utilization was observed in the NAc after sensitization with quinpirole. Importantly these changes were not seen after acute treatment with quinpirole (Carpenter et al., 2003). Additionally, de Haas et al. (2011) have shown alterations in dopamine efflux in the *nucleus accumbens* in the QSM compared to intact animals.

Another brain region of interest in OCD is the OFC. Interestingly, a lesion of the OFC affects the focus of goal-directed activity in compulsive checking (Dvorkin et al., 2010). OFC hyperactivity is considered one of the most prominent intermediate phenotypes of OCD (Ursu and Carter, 2010). Additional interesting results have come from another article by Dvorkin et al. (2008), which showed that hypophysectomy resulting in complex neuro-humoral dysbalances attenuated the locomotor sensitization of quinpirole. Notably it had no effect upon compulsive checking in sensitized animals.

Additionally, it has been shown (Schmidt et al., 2013) that quinpirole sensitization also increased the rewarding effects of *d*-amphetamine. This is important because a link between changes in reward systems, impulsivity and psychiatric disorders including OCD has been convincingly documented (Eslami-Shahrbabaki et al., 2015; Grassi et al., 2015). An important implication of the quinpirole sensitization in relation to neurobiology is a proposal that OCD as a clinical condition can represent a disorder of a “security motivation” system (Szechtman and Woody, 2004). This is a special motivational system of the brain responsible for anticipating potential, but uncertain, future life-threatening situations.

Based on the above mentioned results, we predict that in the QSM, other specific neurobiological changes will be shown. These may include increased connectivity between selected parts of cortico-striatal loops and altered neurotransmission, which may result in deeper attractor basins, defined by a computational stochastic framework of dynamic network theory (Rolls, 2012).

## Predictive Validity of the QSM

### Serotonin-Reuptake Inhibitors and Non-Specific Drugs

A widely used pharmacotherapeutic approach for OCD today is the administration of serotonin-reuptake inhibitors (SRIs) and some relatively non-specific drugs such as tricyclic antidepressants (TCA; e.g., clomipramine; CMI). CMI (Cartwright and Hollander, 1998) is a TCA that acts as a serotonin reuptake inhibitor and antagonist/inverse agonist of histamine H1 receptors, muscarinic acetylcholine receptors, alpha1 adrenergic receptors and dopamine D2 receptors. Effectiveness of SRIs and TCA in OCD triggered studies on the involvement of the serotonergic system in OCD pathophysiology. CMI treatment is effective in only approx. 50% of patients (Leonard et al., 1989). In the QSM, CMI co-administered with quinpirole attenuated compulsive checking (Szechtman et al., 1998). CMI is also effective in reversing another type of OCD-related behavior in quinpirole-sensitized rats—the aforementioned contrafreeloading (Amato et al., 2008; De Carolis et al., 2011). However, the original work on the attenuating effect of CMI demonstrated that this action was transient (Szechtman et al., 1998). Moreover, our new findings show a negative effect of CMI in the QSM on acquisition of spatial learning in an active place avoidance task on a rotating arena (Carousel; Hatalova et al., 2016). This study suggests that adverse effect of CMI on learning the spatial relationships in the QSM might represent a possible interpretation of CMI’s effect on object checking—if animals in the QSM treated with CMI could not learn the spatial associations of places or objects, they could not show the path stereotypies in relation to these objects. Together, these findings provide somewhat contradictory evidence and fail to unequivocally support predictive validity of the QSM of OCD in relation to CMI.

Rather than the TCA clomipramine, first-line treatments for OCD are now selective serotonin reuptake inhibitors (SSRIs), often combined with cognitive-behavioral therapy (CBT). The introduction of SSRIs represented great progress in the treatment of OCD, since they produce fewer side-effects than CMI. The most commonly used SSRIs are fluoxetine, fluvoxamine and paroxetine. Unfortunately, there are limits to the therapeutic efficiency of serotonin reuptake inhibitors. It has been found that approximately 40% of OCD patients do not show an improvement in symptoms following SSRI monotherapy (Skoog and Skoog, 1999; Pallanti et al., 2002). If this first attempt fails then different pharmacological procedures can be tried. Possibilities include changes in dosage (Ninan et al., 2006), route/method of drug administration (Fallon et al., 1998), switching to another serotonin reuptake inhibitor (Fineberg et al., 2015) and addition of neuroleptic drugs (McDougle et al., 1994; Hollander et al., 2003).

Surprisingly, the QSM has never been validated with SSRIs treatment. This stands a bit in contrast with some other models, including behavioral models such as compulsive lever-pressing or genetics models. Only one study tested fluoxetine in the QSM. It has shown that chronic administration of fluoxetine had potentiating effect on locomotor hyperactivity induced by quinpirole, but did not have any effect on stereotyped behavior in the QSM (Collu et al., 1997). Repetitive behavior induced by repeated optogenetic stimulation of cortico-striatal circuits was, however, sensitive to fluoxetine (Ahmari et al., 2013).

### Antipsychotic Drugs

In some patients, co-administration of SSRIs or TCA with classical or atypical neuroleptic drugs improves the efficiency of treatment. Haloperidol and risperidone are the most often used antipsychotics for augmenting SSRI treatment in OCD (McDougle et al., 1994; Hollander et al., 2003). Our data obtained in the QSM suggest a beneficial effect of risperidone added to CMI on performance in an active place avoidance task in the Carousel (Hatalova et al., 2016). However, administration of other antipsychotic drugs such as haloperidol or aripiprazole (dopamine stabilizer) did not improve other OCD-like symptoms (contrafreeloading and hypodipsia) in the QSM (De Carolis et al., 2011). It should be noted that not enough data is available on the effects of antipsychotic drugs in the QSM as there are issues of dosing and schedule that had not been addressed yet.

### Other Pharmacological Substances and Future Challenges

Studies suggest new approaches for cases with inadequate treatment response to SSRI and/or cognitive behavioral therapy. These include augmentation not only with the above- mentioned antipsychotics, but also use of novel drugs acting on glutamate or acetylcholine systems. Nicotine, the agonist of the nicotinic acetylcholine receptor, has been shown to reduce compulsions in OCD patients (Salín-Pascual and Basañez-Villa, 2003; Lundberg et al., 2004) as well as checking in quinpirole-sensitized animals (Tizabi et al., 2002), supporting the predictive validity of this model.

### Deep Brain Stimulation and Neurosurgery

In very serious treatment-resistant OCD cases, deep brain stimulation (DBS) or lesions of brain structures within the cortico-striatal loops may be indicated. DBS is based on high-frequency stimulation of a subcortical brain region, which has an inhibitory effect on nerve tissue and mimics the effect of permanent lesion to some extent (Bourne et al., 2012). The main target structures in OCD patients are the anterior limb of the internal capsule, *nucleus accumbens* (NAc), ventral capsule/ventral striatum, and subthalamic nucleus (STN; Kohl et al., 2014). Of these, the DBS of the NAc and STN has been tested in the QSM. DBS of the NAc shell and core (Mundt et al., 2009) as well as the STN (Winter et al., 2008) decreased checking behavior in the QSM. High-frequency stimulation of globus pallidus and entopeduncular nucleus also reduced checking in the QSM (Djodari-Irani et al., 2011).

The most invasive and irreversible method for treating highly refractory OCD patients is neurosurgical treatment. Significant improvements in patient conditions have been described in anterior cingulotomy, anterior capsulotomy and others (Mindus and Jenike, 1992). The procedure mechanism lies in the disruption of reciprocal connections between cortical areas and subcortical structures. Surprisingly, a reduction in symptom severity is observed with a delay of 3–6 months (Doshi, 2009). Studies with the QSM have revealed that a lesion to the NAc increased checking behavior in saline-treated rats while it did not abolish compulsive checking. Instead, it increased vigor of motor performance (Dvorkin et al., 2010). It is possible that the NAc may be a site for the negative feedback control of checking. The effects of lesions or inactivations of other brain structures involved in OCD circuits in QSM remain yet to be fully understood. The ACC is of particular interest in this context. Functional hyperactivity and decreased volume in this area has been shown repeatedly by converging evidence from different methods (Kopřivová et al., 2011, 2013a,b) yet no study has been aimed to assess the effects of cingulotomy in the QSM.

Together, this suggests that quinpirole exerts its effect on checking behavior by inhibiting the NAc. Notably, a recent study showed that lesion of the NAc did not prevent the development of compulsive checking in the QSM. It only reduced the speed in which checking developed (Ballester González et al., 2015). Interestingly, a recent human DBS study in OCD patients showed that NAc-DBS reduced low-frequency EEG oscillations recorded over the frontal cortex during symptom provocation as well as resting-state functional connectivity (fMRI) between NAc and the prefrontal cortex (Figee et al., 2013a). Our previous as well as other studies reported medial frontal low frequency EEG excess in OCD patients (e.g., Kopřivová et al., 2011, 2013a,b). These findings support the predictive validity of the QSM because similar to DBS, quinpirole sensitization probably inhibits NAc. It would be interesting to test if EEG changes in terms of increased low-frequency bands in the frontal cortex are seen in the QSM. If so, it would be interesting to see how these changes in scalp-recorded EEG relate to intracranial EEG signals and to other functional and structural brain changes. Finally, it would be interesting to test if these potential EEG changes may predict responses to various kinds of treatment. Such electrophysiological studies on the QSM would be particularly useful and highly clinically relevant as scalp-recorded EEG is a cheap, non-invasive and widely available diagnostic method.

## Conclusion

OCD represents a significant challenge for neuropsychiatrists, psychologists, neuroscientists and biomedical researchers in general. For detailed explanations of the brain pathophysiology of this disorder, the QSM could provide valuable insights. The present review discusses the QSM and shows that it has substantial face and construct validity. Evidence on the predictive validity in relation to pharmacotherapy is more contradictory and ambiguous, despite effects of invasive treatments such as DBS and lesions being supportive. In our research, we propose to use the model to continue the search for OCD-related neurobiological and neurochemical changes.

## Author Contributions

AS, JK, DR, and JH wrote major parts of the article. All other authors critically reviewed and edited the article. The review was written based on the expertise of the authors, who have sourced the article on PubMed and Google Scholar.

## Funding

This study was supported by research grant AZV 15-34524A provided by the Ministry of Health, Czech Republic. All rights reserved. Institutional support for Institute of Physiology was provided by RVO: 67985823. Institutional support for NIMH was provided by the project National Institute of Mental Health (NIMH-CZ) grant number ED2.1.00/03.0078 (and the European Regional Development Fund and by the project “Sustainability for the National Institute of Mental Health”, under grant number LO1611, with financial support from the Ministry of Education, Youth and Sports of the Czech Republic under the NPU I program.

## Conflict of Interest Statement

The authors declare that the research was conducted in the absence of any commercial or financial relationships that could be construed as a potential conflict of interest.

## Abbreviations

ACC: Anterior cingulate cortex
CMI: Clomipramine
DBS: Deep brain stimulation
fMRI: Functional magnetic resonance imaging
NAc: *Nucleus accumbens*
OCD: Obsessive-compulsive disorder
OFC: Orbitofrontal cortex
QSM: Quinpirole sensitization model
SRIs: Serotonin reuptake inhibitors
SSRIs: Selective serotonin re-uptake inhibitors
STN: Subthalamic nucleus
TCA: Tricyclic antidepressants.
